# A New Crystal Structure of the Bifunctional Antibiotic Simocyclinone D8 Bound to DNA Gyrase Gives Fresh Insight into the Mechanism of Inhibition

**DOI:** 10.1016/j.jmb.2014.02.017

**Published:** 2014-05-15

**Authors:** Stephen J. Hearnshaw, Marcus J. Edwards, Clare E. Stevenson, David M. Lawson, Anthony Maxwell

**Affiliations:** Department of Biological Chemistry, John Innes Centre, Norwich Research Park, Norwich NR4 7UH, UK

**Keywords:** ITC, isothermal titration calorimetry, SD8, simocyclinone D8, ASU, asymmetric unit, EDTA, ethylenediaminetetraacetic acid, DNA gyrase, DNA topoisomerase, antibiotics, X-ray crystallography, simocyclinones

## Abstract

Simocyclinone D8 (SD8) is an antibiotic produced by *Streptomyces antibioticus* that targets DNA gyrase. A previous structure of SD8 complexed with the N-terminal domain of the DNA gyrase A protein (GyrA) suggested that four SD8 molecules stabilized a tetramer of the protein; subsequent mass spectrometry experiments suggested that a protein dimer with two symmetry-related SD8s was more likely. This work describes the structures of a further truncated form of the GyrA N-terminal domain fragment with and without SD8 bound. The structure with SD8 has the two SD8 molecules bound within the same GyrA dimer. This new structure is entirely consistent with the mutations in GyrA that confer SD8 resistance and, by comparison with a new apo structure of the GyrA N-terminal domain, reveals the likely conformation changes that occur upon SD8 binding and the detailed mechanism of SD8 inhibition of gyrase. Isothermal titration calorimetry experiments are consistent with the crystallography results and further suggest that a previously observed complex between SD8 and GyrB is ~ 1000-fold weaker than the interaction with GyrA.

## Highlights

• Fragment engineered to reveal biologically relevant structure of GyrA–drug complex.
• This structure fully explains all available biochemical/biophysical/genetic data.
• Binding site in GyrB is ~ 1000-fold weaker than site in GyrA.

## Graphical abstract

**Figure:**
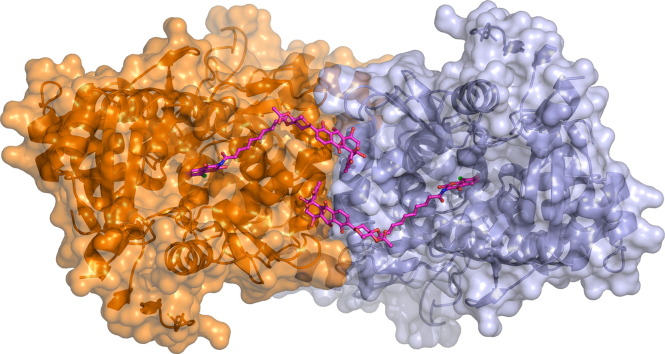

## Introduction

DNA topoisomerases are enzymes that control the topological state of DNA in cells [1–3]. These enzymes are divided into two types depending on whether their mechanism of action involves breaking one (type I) or both (type II) strands of DNA. DNA topoisomerases are capable of performing DNA supercoiling and relaxation, catenation and decatenation, and knotting and unknotting, as a result of their ability to pass one segment of DNA through a break in another. DNA gyrase is the only type II topoisomerase capable of catalyzing DNA supercoiling, in a reaction driven by ATP hydrolysis [4]. DNA gyrase consists of two subunits, GyrA and GyrB (97 kDa and 90 kDa, respectively, in *Escherichia coli*), forming an A_2_B_2_ complex in the active enzyme. The N-terminal domain of GyrB is the site of ATP hydrolysis, while DNA cleavage and strand passage occur at the interface between the C-terminal domain of GyrB and the N-terminal domain of GyrA. This interface is known as the “DNA gate”; a second interface in GyrA via which the passed DNA segment leaves the protein is termed the “exit gate” [5,6].

Crucially, DNA gyrase is essential for all bacteria, but not found in humans, making it an ideal target for antibiotics [4,7,8], as illustrated by the highly successful fluoroquinolones [9,10]. Aminocoumarins, such as novobiocin and clorobiocin, are another class of well-characterized DNA gyrase inhibitors [11,12] but are less clinically successful because of toxicity and solubility issues. Simocyclinone D8 (SD8) is a bifunctional antibiotic isolated from *Streptomyces antibioticus* Tü 6040 [13–16] that consists of a chlorinated aminocoumarin moiety linked to an angucyclic polyketide via a tetraene linker and a d-olivose sugar. In contrast to other aminocoumarins, such as novobiocin, that bind to the GyrB N-terminal domain and inhibit ATPase activity, SD8 binds to the GyrA N-terminal domain and prevents the binding of DNA [17], although there is some evidence that SD8 can also bind to the C-terminal domain of GyrB [18].

The interaction between DNA gyrase and SD8 has been well studied [17–21], including the crystal structure of a complex formed between a 59-kDa N-terminal domain (NTD) fragment of the *E. coli* gyrase A subunit (GyrA59) and SD8 [21]. This structure revealed two binding pockets that separately accommodate the aminocoumarin and polyketide moieties of SD8, forming a cross-linked GyrA59 tetramer in the crystal. The existence of the tetrameric species was identified in solution by both analytical ultracentrifugation and mass spectrometry under similar protein-to-ligand ratios as those used to obtain crystals, confirming that it was not simply a crystallographic artifact [21]. Further analysis using mass spectrometry showed that the major solution-state species at lower ligand concentrations is the GyrA59 dimer with one or two SD8 ligands bound, leading to the proposition (and modeling) of SD8 binding to GyrA59 in an alternative conformation that makes use of binding pockets within a GyrA homodimer rather than bridging between dimers [18]. The present work tests the validity of this modeled binding conformation and sheds new light on the mechanism of inhibition of DNA gyrase by SD8.

## Results and Discussion

### Construction and properties of a 55-kDa GyrA N-terminal domain fragment

A new GyrA NTD fragment was constructed with a view of obtaining a crystal structure of SD8 bound to GyrA in an alternative conformation that would be representative of the situation at lower ligand concentrations. This smaller 55-kDa NTD fragment of GyrA (GyrA55), comprising residues 30–522 of the 875-amino-acid wild-type sequence (compared with residues 2–522 for GyrA59), crucially lacks residues spanning Leu17 to Asp23 that have been identified as providing 10 of the 12 protein–protein hydrogen bonds responsible for stabilizing the tetramer at the dimer–dimer interface in the crystal structure of the GyrA59–SD8 complex [21]. This 55-kDa fragment also lacks the N-terminal α-helix (α-helix 1), but to allow easier comparisons with the work on the 59-kDa fragment, the same numbering of α-helices is used; that is, the N-terminal helix in the GyrA55 structures is referred to as α-helix 2.

The circular dichroism (CD) spectrum of GyrA55 confirmed that the protein is folded and is consistent with the spectrum obtained for GyrA59 (Fig. S1). Isothermal titration calorimetry (ITC) showed that GyrA55 is still capable of binding SD8, yielding binding parameters that are consistent with those previously determined for GyrA59 (Fig. S2) [17].

### Crystal structures of apoGyrA55 and the GyrA55–SD8 complex

**Fig. 1:**
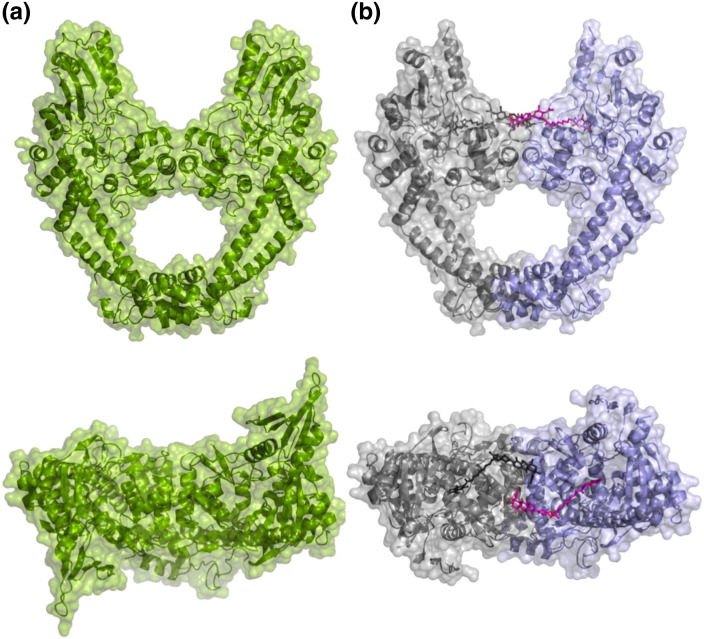
Orthogonal views of biological dimers of the GyrA55 crystal structures presented in this work. (a) Two of the four monomers of apoGyrA55 that comprise the ASU. (b) One monomer from the ASU of the GyrA55–SD8 complex (blue) with the crystallographic symmetry-related molecule with which it forms the biological dimer (gray). The proteins are depicted in cartoon representation with a semitransparent surface and the SD8 molecules are shown as magenta or black sticks in the ASU or symmetry-related monomer, respectively.

The 1.9-Å-resolution apoGyrA55 structure contains four GyrA55 monomers in the asymmetric unit (ASU) (Fig. S3), forming two biologically relevant homodimers, an example of which can be seen in Fig. 1a. Comparisons within this structure reveal some conformational flexibility, with overall RMSD values up to 1.28 Å for monomer–monomer superpositions and up to 1.37 Å for dimer–dimer superpositions. Superposition of each homodimer with a homodimer generated from the apoGyrA59 [22] crystal structure (which has a single monomer in the ASU) gives RMSD values of 0.87 and 1.20 Å, indicating that there is less variation across these two structures than within the apoGyrA55 structure.

**Fig. 2:**
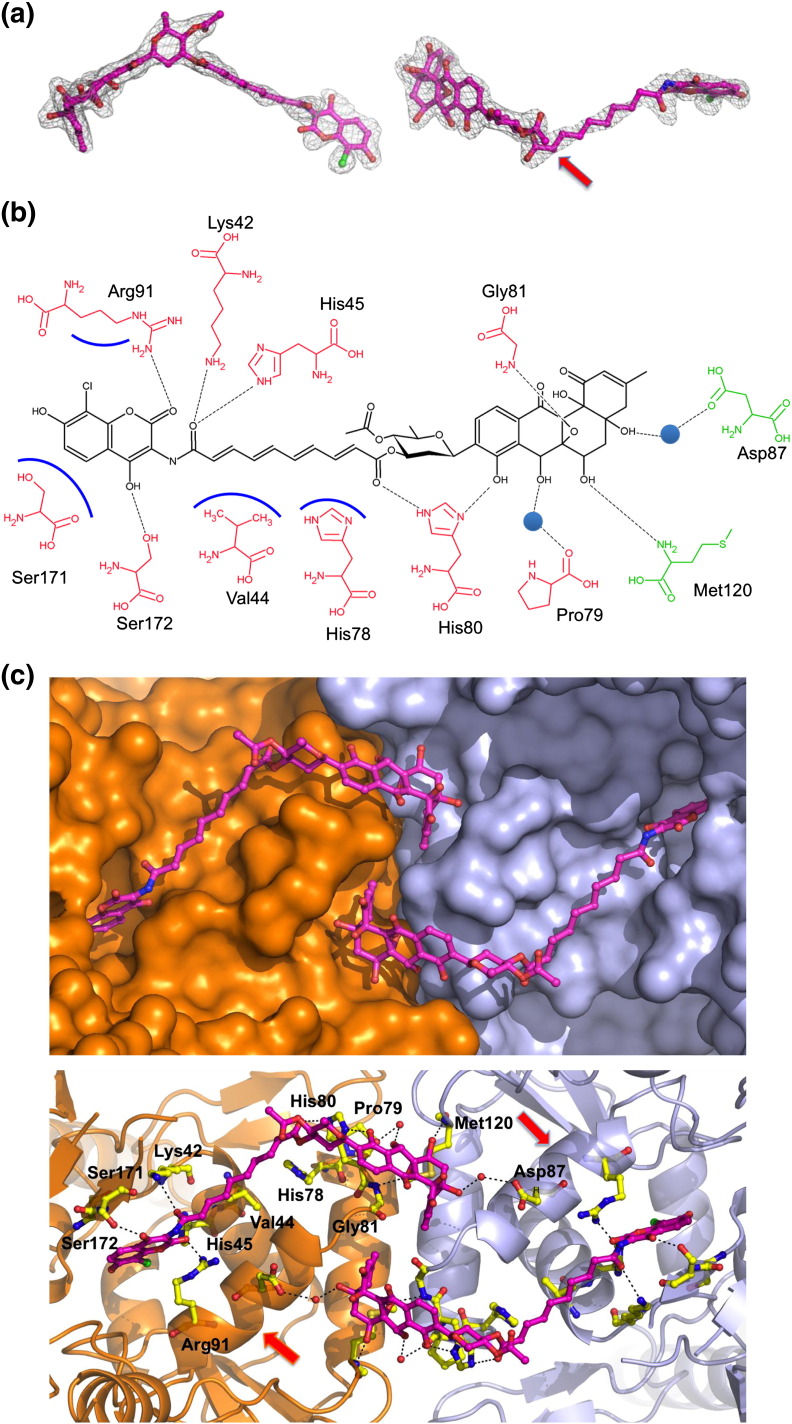
Details of the SD8-binding site. (a) Orthogonal views of simulated annealing omit electron density map for SD8 contoured at 1.5 σ and superposed on the final coordinates of the ligand. The red arrow indicates the position of the *cis* bond in the SD8 tetraene linker. (b) Schematic figure detailing protein–ligand interactions in the GyrA55–SD8 complex. Broken lines represent hydrogen bonds, blue lines represent residues with hydrophobic interactions, and blue circles represent water molecules. SD8 is colored black, and residues from the two monomers are colored red and green, respectively. (c) Close-up of the GyrA55–SD8-binding sites, the upper panel displays the protein with a surface representation to highlight the shape of the binding pockets, while the lower panel shows the specific residues that bind SD8. Residues are depicted as yellow sticks, associated water molecules as red spheres, and hydrogen-bonding interactions with black broken lines and red arrows indicate the position of helix 4 in each monomer.

The 2.05-Å-resolution GyrA55–SD8 complex structure contains two GyrA55 monomers in the ASU, although they do not form the biologically relevant homodimer. Instead, two distinct homodimers can be generated from these monomers by the addition of crystallographic symmetry-related monomers (Fig. S4). One of the resultant homodimers (Fig. 1b) binds two crystallographically equivalent SD8 molecules in a novel conformation (Fig. 2). However, SD8 binding to the second homodimer is compromised by crystal packing; the first homodimer would otherwise clash with the olivose moiety of a similarly bound SD8 molecule. Nevertheless, there is some positive difference electron density in the vicinity of the aminocoumarin-binding pocket of dimer 2, suggestive of a partially ordered SD8 ligand (Fig. S5). The two monomers within the ASU are closely superposable (overall RMSD value of 0.33 Å), while the crystallographically generated dimers show larger differences (overall RMSD value of 1.23 Å), which is perhaps largely a consequence of variations in the flexing of the long α-helices of the coiled-coil domains that link the exit and DNA gates. The subsequent analysis will focus mainly on the fully ligand-bound dimer.

**Fig. 3:**
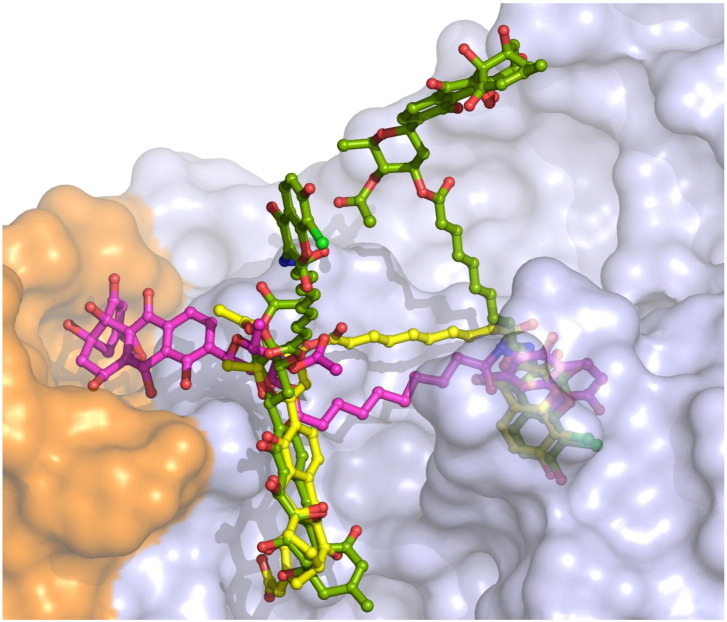
A comparison between the intra-dimer binding conformations of SD8 from the X-ray structure of the GyrA55–SD8 complex presented here (magenta), the inter-dimer SD8 binding conformation from the X-ray structure of the GyrA59–SD8 complex (green; two SD8 molecules shown) [21], and the proposed intra-monomer binding model that links the polyketide and aminocoumarin-binding sites that were observed in the same monomer of the latter X-ray structure (yellow) [18]. In the GyrA59–SD8 complex structure, the protruding polyketide and aminocoumarin moieties are accommodated in binding pockets from a second GyrA59 homodimer (not shown). The semitransparent protein surface corresponds to the GyrA55–SD8 complex crystal structure.

The conformation of the SD8 in GyrA55–SD8 is significantly different to that observed in the previous study [21], principally because it is bound entirely within one GyrA55 homodimer. The polyketide moiety has shifted to a position where it spans the interface between the two GyrA monomers, with SD8 forming hydrogen-bonding interactions with residues from each monomer, specifically as follows: direct hydrogen-bonding interactions with His80, Gly81, and Met120 (from the adjacent monomer) and indirect hydrogen-bonding interactions (via water molecules) with Pro79 and Asp87 (from the adjacent monomer) (Fig. 2b and c). The aminocoumarin-binding pocket is essentially the same as that observed for the GyrA59–SD8 complex, but its orientation in the binding pocket is different (Fig. 3). The aminocoumarin moiety is bound to the protein through hydrogen-bonding interactions with Lys42, His45, Arg91, and Ser172 and hydrophobic contacts with Val44. When overlaid, the tetraene linkers of the GyrA59–SD8 and GyrA55–SD8 complex structures exit the aminocoumarin pocket at an angle of ~ 70° with respect to one another (Fig. 3). In the latter, the tetraene linker actually follows a similar path to that of the intra-dimer conformation proposed in the previous study [21], although it is more remote from α-helix 4 and does not interact with it at all (Fig. 2c). Additionally, there are no lobes of additional electron density adjacent to the polyketide moiety, which were modeled as Mg^2 +^ in the GyrA59–SD8 complex. In order to accommodate SD8 in this new binding configuration, a single *cis* bond is required in the tetraene linker adjacent to the ester linkage with the olivose and is fully consistent with the observed electron density (Fig. 2a). This *cis* bond introduces a distinctive kink in the antibiotic such that the polyketide moieties of the symmetry-related molecules are directed toward one another giving a closest interatomic distance of only 4.6 Å.

**Fig. 4:**
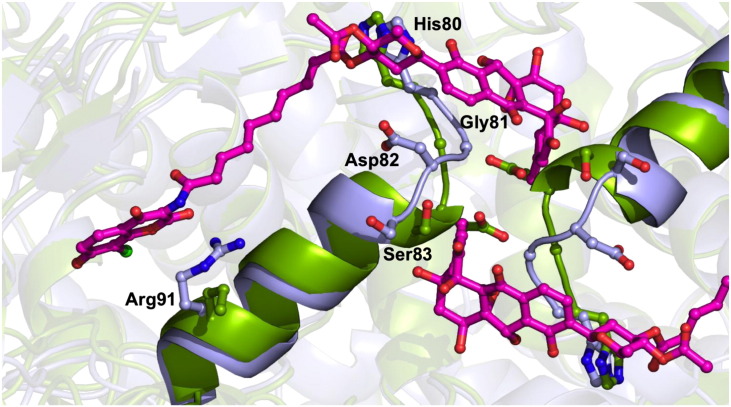
Conformational changes at the SD8-binding sites between GyrA55–SD8 and apoGyrA55. Specifically, the movement of Arg91 in the aminocoumarin pocket of GyrA55–SD8, the partial unwinding of helix 4, and conformational changes in the following loop in GyrA55–SD8 (His80–Ser83) that prevent clashes and aid binding with the polyketide moiety. GyrA55–SD8 and apoGyrA55 are colored blue and green, respectively; SD8 molecules are represented by magenta sticks, and the side chains of key residues from each structure are represented by sticks and helices 4 as filled cartoons.

Superposition of each GyrA55–SD8 with each apoGyrA55 dimer gives overall RMSD values below 1.44 Å, while values for all possible monomer–monomer comparisons did not exceed 1.07 Å, indicating that there are no major conformational changes in the 55-kDa GyrA fragment upon SD8 binding. The only significant change is that the N-terminal end of α-helix 3 partially unwinds (it becomes two residues shorter), allowing the protein main chain in this region to move in order to accommodate the polyketide moiety of one SD8 molecule and, in so doing, projects the side chain of Asp87 toward the symmetry-related SD8 molecule, such that it hydrogen bonds, via a water molecule, to the ester group connecting the tetraene linker to the olivose sugar. As part of this backbone rearrangement, the plane of the His80–Gly81 peptide bond twists through ~ 90° to allow hydrogen bonding of the main chain amide of Gly81 to the epoxide group of the polyketide moiety in the same SD8 molecule. Additionally, there are a number of side-chain rearrangements around the SD8-binding site, but most of these are relatively minor, the exception being Arg91, which is disordered in the apoGyrA55 structure, with either no clear density for the guanidinium group and/or high temperature factors in each of the four monomers, suggesting that it is highly mobile in the absence of SD8 (Fig. 4).

### Re-evaluation of GyrA mutant data

In the previous crystallographic study of the GyrA–SD8 interaction [21], a number of point mutants were generated to probe the protein–ligand interaction. The effects of many of these mutations could be rationalized with reference to the structure of the GyrA59–SD8 complex, but others could not be adequately explained. These data were re-evaluated in the context of the GyrA55–SD8 complex structure.

Mutations to Arg32, Arg47 (identified as binding the polyketide moiety of SD8 in the GyrA59–SD8 complex crystal structure), and Asn165 (identified as binding the aminocoumarin moiety of SD8 in the GyrA59–SD8 complex crystal structure) had either no increase in resistance to SD8 in DNA supercoiling assays or no change in binding characteristics, as determined by surface-plasmon resonance, when compared to wild-type GyrA [21]. The change in the SD8 orientation in GyrA55–SD8 repositions the polyketide moiety away from Arg32 and Arg47 (Arg47 is 6.3 Å from SD8); it also moves the aminocoumarin moiety away from Asn165 (Asn165 is 6.6 Å from SD8), making any interactions with any of these residues very unlikely.

Conversely, mutations to Gly81 and Asp87 were found to confer resistance to SD8 (identified by sequencing SD8-resistant mutants) despite no clear role for these residues in the stabilization of SD8 in the GyrA59–SD8 complex crystal structure. These mutations are readily interpretable with reference to the GyrA55–SD8 structure, since Gly81 makes direct contact with the polyketide moiety of SD8 and Asp87 makes indirect contact with the polyketide moiety of the symmetry-related SD8 molecule via a water molecule (Fig. 2b and c).

To further test the validity of the intra-dimer binding mode of SD8 observed in the GyrA55–SD8 complex, we made three additional potentially disruptive mutations: M120P in the polyketide pocket, K42A in the aminocoumarin-binding pocket, and A84R in α-helix 4 that runs parallel with the tetraene linker. The M120P and K42A mutants resulted in 60- and 50-fold increases in resistance to SD8, respectively, which tallies with the loss of key hydrogen bonds. Conversely, the A84R mutant showed little or no increase in resistance to SD8, but this could be rationalized by the longer side chain adopting a conformation that does not impinge on the SD8-binding site. Sample data are shown in Fig. S6; a full analysis of how each mutant affects the GyrA–SD8 complex is given in Table 1. Overall we conclude that the SD8 orientation observed in the GyrA55–SD8 structure is fully representative of the *in vitro* (and likely the *in vivo*) binding mode of SD8 to gyrase at physiologically relevant concentrations due to the strong correlation with all the available mutant data.

### Comparison with existing type II topoisomerase structures leading to a proposition of the inhibitory mechanism of SD8

Wendorff *et al.* analyzed all 22 type II topoisomerase structures available in the Protein Data Bank at the time of publication and found that there were 6 distinct sub-groups based on the exit-gate position, as well as the binding of DNA or drug molecules [23]. The structures presented in this work fit with the group of type II topoisomerase structures that have a closed exit gate and no DNA bound; that is, they are consistent with the existing structures. Observing the differences between apo and DNA-bound type II topoisomerases in these groupings highlights conserved conformational changes that occur at the DNA gate to facilitate DNA binding.

**Fig. 5:**
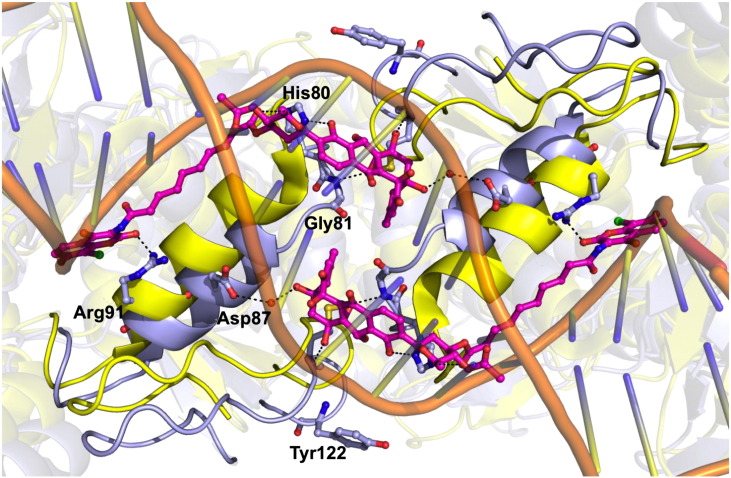
A comparison of the DNA gates from GyrA55–SD8 (light blue) and *Staphylococcus aureus* GyrA in complex with DNA and GSK299423 (ligand not shown) (yellow) [24]. The protein and DNA are depicted in semitransparent cartoon representation, with regions of interest depicted as filled cartoon. Key residues and the SD8 molecules are shown as sticks, with the latter in magenta.

These comparisons also allow us to speculate on the mechanism by which SD8 blocks DNA binding to gyrase, by comparing the GyrA55–SD8 complex structure with gyrase structures in complex with DNA. The positioning of SD8 across the dimer interface effectively “staples” the dimer closed, which not only has the effect of preventing the DNA gate from opening for strand passage but also precludes the observed conformational changes that need to occur around the DNA gate to allow DNA binding. Specifically, the interactions of Asp87 and Gly81 with SD8 would prevent the necessary movement of α-helix 4; the interaction between Met120 and SD8 would prevent the loop containing the catalytic tyrosine (Tyr122) from orienting itself correctly with respect to the DNA, and the interaction of Arg91 (also found on α-helix 4) with the aminocoumarin moiety of SD8 would prevent Arg91 from stabilizing the GyrA–DNA complex (Fig. 5).

### ITC analysis of SD8 binding to gyrase

ITC analysis was carried out to further characterize the binding properties of SD8 to gyrase. An interaction was observed when SD8 was injected into a GyrA55 solution, yielding a binding constant of 44 nM and indicating an approximate 1:1 stoichiometry (Fig. S2a). These parameters are consistent with those previously determined for GyrA59 [17].

Work by Sissi *et al*. suggested a new SD8-binding site in GyrB [20]. This was based on the results of CD experiments, which showed that SD8 affects the thermal transitions of GyrB as well as GyrA, and proteolytic digestion studies, which found that SD8 affects the proteolysis pattern resulting from the digestion of GyrB47 (the C-terminal domain of GyrB) by trypsin. We have used ITC to further explore the binding of SD8 to GyrB.

An interaction was observed when SD8 was injected into a GyrB solution (data not shown). An interaction was not observed with GyrB43 (N-terminal GyrB domain) but was observed with GyrB47 (C-terminal GyrB domain), yielding a binding constant of 46 μM and indicating an approximate 1:1 stoichiometry (Fig. S2b). This finding is consistent with that of Sissi *et al.*, who observed an SD8-binding site in the C-terminal domain of GyrB [20]. However, the substantially higher affinity of SD8 for GyrA over GyrB (approximately 1000-fold in this study) would suggest that GyrA is the primary target for SD8 and that the interaction with GyrB may be an *in vitro* artifact that is only manifested in the absence of GyrA. These observations may correlate with the promiscuous binding we have observed with the polyketide moiety of SD8 in the GyrA59–SD8 and GyrA55–SD8 complex structures.

### Conclusion and summary

In this work we have presented a new structure for an N-terminal GyrA–SD8 complex (GyrA55–SD8) that is significantly different to that observed in the previous study [21], principally because the antibiotic is bound entirely within one GyrA55 homodimer. We have shown, through evaluation of mutant data, that this new structure is more likely to be representative of the mode of action of SD8 on DNA gyrase and, through comparisons with existing type II topoisomerase structures, provides a molecular level explanation of the mechanism of action of SD8. In addition, we have also presented the high-resolution structure of apoGyrA55 that has allowed us to describe specific conformational changes brought on by the action of SD8 binding to gyrase.

## Materials and Methods

### Expression and purification of proteins

The N-terminal 55-kDa fragment (exact molecular mass, 55,390.5 Da) of *E. coli* DNA gyrase subunit A (GyrA55), comprising residues 30–522 of the 875-amino-acid wild-type sequence (UniProtKB/Swiss-Prot entry P0AES4), and the GyrA59 protein were produced using a modification of the previously published procedure [21,25]. GyrA mutants and GyrB and GyrB fragments were expressed and purified as described previously [26–28].

### Crystallization and X-ray data collection

A purified GyrA55 sample, at a concentration of 10 mg/ml in TGED buffer [50 mM Tris–HCl (pH 7.5), 1 mM ethylenediaminetetraacetic acid (EDTA), 1 mM DTT, and 10% (v/v) glycerol], was used for crystallization. For the apoGyrA55 structure, crystals were grown in hanging drops composed of 1.5 μl protein solution and 1.5 μl reservoir solution [0.1 M Tris–HCl (pH 8.5) and 30% (v/v) polyethylene glycol 300] equilibrated against 1 ml reservoir solution at 20 °C. The crystals were mounted in LithoLoops (Molecular Dimensions) and cooled by plunging into liquid nitrogen without the need for further cryoprotection. For the GyrA55–SD8 structure, crystals were grown in sitting drops composed of 0.3 μl protein solution and 0.3 μl reservoir solution [0.1 M 4-morpholineethanesulfonic acid (pH 6.5) and 40% (v/v) polyethylene glycol 200] equilibrated against 50 μl reservoir solution at 20 °C. Prior to mounting, crystals were soaked overnight in the crystallization solution, with the addition of 1 mM SD8 in 10% (v/v) DMSO (final concentration) and could also be cooled by plunging into liquid nitrogen without the need for further cryoprotection. Diffraction data were recorded at the Diamond Light Source on beamline i24 with a Pilatus 6M detector for the apoGyrA55 structure and on beamline i04-1 with a Pilatus 2M detector for the GyrA55–SD8 structure. The resultant data sets were processed using Xia2 [29] and the statistics are summarized in Table 2.

### Structure determination and refinement

A monomer from the existing 2.6-Å-resolution crystal structure of GyrA59–SD8 complex (PDB accession code 2Y3P [21]) was used as a search model for molecular replacement with the apoGyrA55 data after stripping away all solvent molecules and the ligand. Molecular replacement was performed using Phaser [30]. Four independent molecules were located in the ASU. Solvent content estimations based on four GyrA55 monomers per ASU gave a value of 59.0%. Manual rebuilding of this initial model was performed with Coot [31], and this was alternated with cycles of restrained refinement with REFMAC5 [32]. Non-crystallographic asymmetry restraints were employed and, in the final stages, TLS refinement was used with a total of 24 TLS domains, which were defined using the TLS motion determination server† [34]. The statistics of the final model are summarized in Table 2.

A monomer from this 1.9-Å-resolution crystal structure of apoGyrA55 was used as a search model for molecular replacement with the GyrA55–SD8 data after stripping away all solvent molecules. Two independent molecules were located in the ASU (these monomers did not form the biological dimer, but the biological dimers could be generated via crystallographic symmetry), and solvent content estimations based on two GyrA55 monomers per ASU gave a value of 57.8%. The same refinement techniques were applied to this data set, and a total of 16 TLS domains were used in the final stages of the procedure. Residual electron density was apparent for one complete SD8 molecule in one monomer of the ASU, and some density was seen in the vicinity of the aminocoumarin pocket of the other monomer, but this was not well resolved and consequently was left vacant. The statistics of the final model are summarized in Table 2.

### Mutagenesis

Site-directed mutants were made in full-length GyrA using the Phusion Site-Directed Mutagenesis kit (Thermo Scientific) according to the manufacturer's protocol, using plasmid pPH3 [35]. The mutations were confirmed by sequencing (Genome Enterprise Ltd.).

### DNA supercoiling assay

Gyrase supercoiling assays were performed as described previously [36]. Samples (30 μl) containing gyrase (22 nM) and 0.5 μg of relaxed pBR322 DNA (6 nM) were incubated at 37 °C for 30–90 min (depending on the GyrA used: 90 min for the less active K42A and M120P GyrA mutants; 30 min for all others) in the presence of 1 mM ATP. The DNA was prepared for electrophoresis by the addition of 30 μl of phenol-chloroform-isoamyl alcohol (25:24:1), 15 μl of 40% (w/v) sucrose, 0.1 M Tris–HCl (pH 8), 0.1 M EDTA, and 0.5 mg/ml bromophenol blue, brief vortexing and centrifugation (15,700*g*, 5 min). The blue upper phases of the products were analyzed on 1% (w/v) agarose gels. To determine approximate IC_50_ values for SD8, we carried out supercoiling assays including a range of SD8 concentrations. Gels were analyzed and the IC_50_ determined by visual inspection of the intensity of the supercoiled DNA band in the SD8 containing lanes, relative to the no drug control lane, as described previously [19].

### Isothermal titration calorimetry

Enthalpy values were measured by using an iTC_200_ isothermal titration calorimeter (Microcal, Milton Keynes, United Kingdom). Gyrase subunits and domains (GyrA55, GyrB43, and GyrB47) were dialyzed extensively against binding buffer [50 mM Tris–HCl (pH 7.5), 1 mM EDTA, and 100 mM KCl]. During the titration, the protein (200 μl, at 5–15 μM) was added to the sample cell, and 27 successive aliquots of SD8 (at 100–250 μM) [1.5 μl volumes except for the first one (0.5 μl)] were injected at 2-min intervals. All titrations were carried out at 25 °C. A constant DMSO concentration of 3% (v/v) (obtained by addition of DMSO to protein and SD8 samples) was used to aid SD8 solubility. The upper limit of SD8 concentration was 250 μM due to the poor solubility of the drug at higher concentrations. Data were analyzed by nonlinear regression using a single-site binding model with Origin software (Microcal), which yielded independent values for *K*_d_.

### CD spectroscopy

CD spectroscopy experiments were performed using a Chirascan-Plus CD spectrophotometer (Applied Photophysics). Proteins at concentrations of approximately 10 mg/ml (in TGED buffer) were diluted to 0.25 mg/ml in 20 mM potassium phosphate (pH 7.2). CD measurements were carried out in a quartz glass cuvette with a 0.5-mm path length at 20 °C. Each CD spectrum is the average of 4 scans collected between 190 and 260 nm, using a bandwidth of 2.0 nm, a step size of 0.5 nm, and time points of 1 per second. Secondary structure assignments were made using the DichroWeb server, employing the Cdsstr method with reference set 7 [37,38].

### Accession numbers

Coordinates and structure factors for the apoGyrA55 and GyrA55–SD8 structures described herein have been deposited in the Protein Data Bank with accession codes 4CKK and 4CKL, respectively.

## Figures and Tables

**Table 1:** Summary of SD8 resistance data for GyrA mutants

Type of mutant	Mutation	Relative IC_50_ (supercoiling) compared to wild-type GyrA	Context in GyrA59–SD8	Context in GyrA55–SD8
Resistance predicted based on GyrA59–SD8, but not observed^a^	Arg32 → Ala	Inactive; surface-plasmon resonance shows no change in SD8 binding	H-bond to PK	Absent from model; superposition with GyrA59 indicates that it is remote from SD8
	Arg47 → Ala	No change	2 H-bonds to PK	6.3 Å from TL
	Asn165 → Ala	No change	H-bond to AC	6.1 Å from AC
Resistance not predicted based on GyrA59–SD8, but was observed^a^	Gly81 → Ser	40-fold increase	Close to OL	H-bond to PK
	Asp87 → Tyr	57-fold increase	7.0 Å from TL	H-bond to PK via water
Resistance predicted based on GyrA59–SD8, and observed^a^	His80 → Ala	230-fold increase	Aromatic stacking with PK	H-bond to PK
	His45 → Ala	9-fold increase	Aromatic stacking with AC	H-bond to AC
	Arg91 → Ala	20-fold increase	H-bond to AC	H-bond to AC
Resistance predicted based on GyrA55–SD8, and observed^b^	Met120 → Pro	60-fold increase	Remote from SD8	H-bond to PK
	Ala84 → Arg	2 fold-increase	7.9 Å from TL	4.0 Å from TL
	Lys42 → Ala	50-fold increase	H-bond to TL	H-bond to TL
Quinolone-resistant mutations^a^	Ser83 → Trp	10-fold increase	8.3 Å from PK	Disrupt PK binding
	Ala84 → Pro	38-fold increase	Disruption to α-helix 4	Disruption to α-helix 4

All mutations were made with full-length GyrA. Distances quoted are closest atom–atom contacts. AC, aminocoumarin moiety; PK, polyketide moiety; OL, olivose sugar; TL, tetraene linker.

a Mutants generated and tested in a previous study [21].

b Mutants generated and tested in this work.

**Table 2:** Summary of GyrA55 X-ray data and model parameters

Data set	apoGyrA55	GyrA55–SD8
*Data collection*		
Space group	*P*1	*C*2
Cell parameters		
a, b, c (Å)	93.57, 95.53, 95.89	160.82, 96.05, 112.35
α, β, γ (°)	105.16, 118.81, 103.42	90.00, 132.72, 90.00
Solvent content (%)	59.0	57.6
Beamline^a^	i24	i04-1
Wavelength (Å)	0.9686	0.9173
Resolution range^b^ (Å)	81.51–1.90	41.51–2.05
	(1.95–1.90)	(2.10–2.05)
Unique reflections^b^	194,509 (14,158)	77,820 (5648)
Completeness^b^ (%)	97.0 (94.7)	99.0 (98.1)
Multiplicity^b^	2.9 (2.6)	7.2 (5.4)
*R*_merge_b,c	0.067 (0.486)	0.088 (0.895)
*R*_meas_a,c	0.095 (0.687)	0.094 (0.996)
CC_½_a,d	0.993 (0.598)	0.999 (0.533)
〈*I*〉/〈σ*I*〉^b^	11.0 (2.7)	15.0 (2.0)
Wilson *B* value (Å^2^)	23.6	30.3

*Refinement*		
*R*_cryst_^d^ (based on 95% of data)	0.169	0.196
*R*_free_^d^ (based on 5% of data)	0.189	0.225
Coordinate error estimate^e^ (based on *R*_free_; Å)	0.109	0.146
Ramachandran favored/allowed/disallowed^f^ (%)	98.4/1.4/0.2	97.9/1.4/0.7
RMSD bond distances (Å)	0.013	0.015
RMSD bond angles (°)	1.502	1.643

*Contents of model (molecules/non-hydrogen atoms)*		
Protein	1942/15,016	885/6784
SD8	—	1/66
Water molecules	1056	284

*Average temperature factors (Å^2^)*		
Protein	31.9	42.6
SD8	—	43.1
Waters	37.3	40.7
Overall	32.3	42.6
PDB accession code	4CKK	4CKL

a i24 and i04-1 are beamlines at the Diamond Light Source (Oxfordshire, UK).

b The figures in parentheses indicate the values for the outer-resolution shell.

c *R*_merge_ = ∑*_hkl_*∑*_i_*|*I_i_*(*hkl*) − 〈*I*(*hkl*)〉|∑*_hkl_*∑*_i_I_i_*(*hlk*), where *I_i_*(*hlk*) is the *i*th observation of reflection *hkl* and 〈*I*(*hkl*)〉 is the weighted average intensity for all observations *i* of reflections *hkl*.

d The *R*-factors *R*_cryst_ and *R*_free_ are calculated as follows: *R* = ∑(|*F*_obs_ − *F*_calc_|)/∑|*F*_obs_| × 100, where *F*_obs_ and *F*_calc_ are the observed and calculated structure factor amplitudes, respectively.

e Estimate of the overall coordinate errors calculated in REFMAC5 based on *R*_free_.

f Calculated using MolProbity [33].

## Footnotes

† http://skuld.bmsc.washington.edu/~tlsmd/
