# Supplementary material for: A New Crystal Structure of the Bifunctional Antibiotic Simocyclinone D8 Bound to DNA Gyrase Gives Fresh Insight into the Mechanism of Inhibition
Source: J Mol Biol. 2014 May 15;426(10):2023–33. doi: 10.1016/j.jmb.2014.02.017 (PMC4018983; doi:10.1016/j.jmb.2014.02.017)
Supplement: Supplementary file 1 — Supplementary figures. [file mmc1.docx]

**Supplementary Material**

**Figure S1**

**
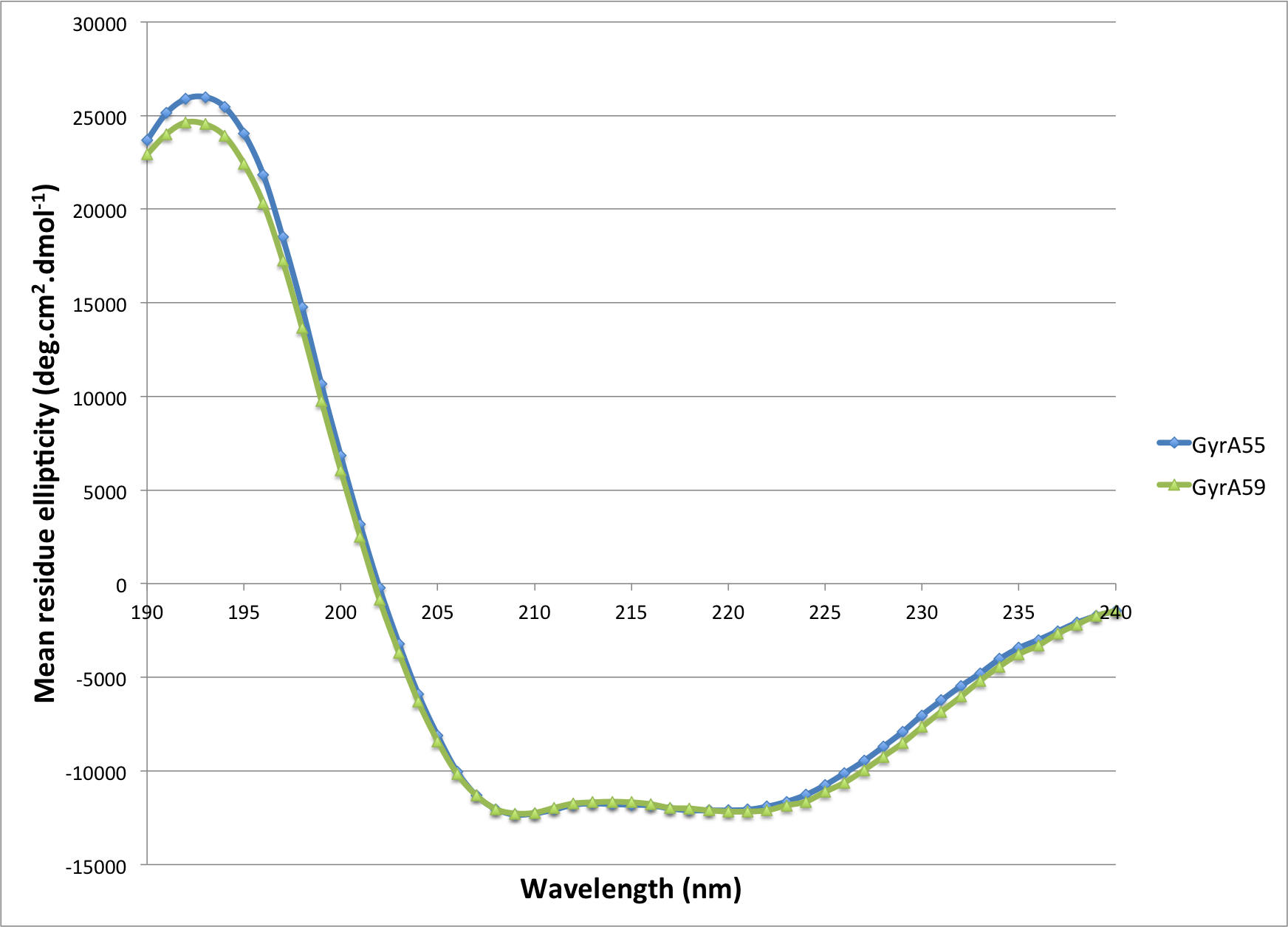
**

**Fig. S1.** Far UV CD spectra of GyrA55 and GyrA59. Each domain has an essentially identical secondary structure composition of: 41% α-helix, 16% β-sheet, 19% turns and 23% other.

**Figure S2**

**(a) (b)**


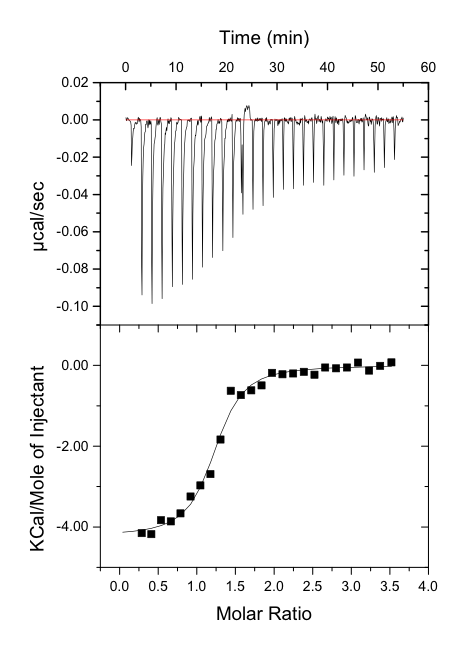
**
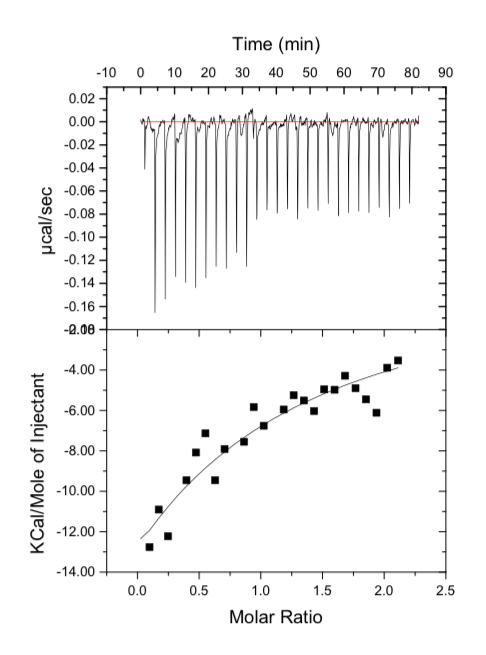
**

**Fig. S2.** Binding of SD8 to **(a)** GyrA55 and **(b)** GyrB47 by isothermal titration calorimetry. The upper panels of the figure present the raw ITC data, while in the lower panels the data are presented as kilocalories per mole of injectant vs. the molar ratio of ligand/protein, where the solid line shows the best fit to a one-site model obtained by least-squares regression.

**Figure S3**


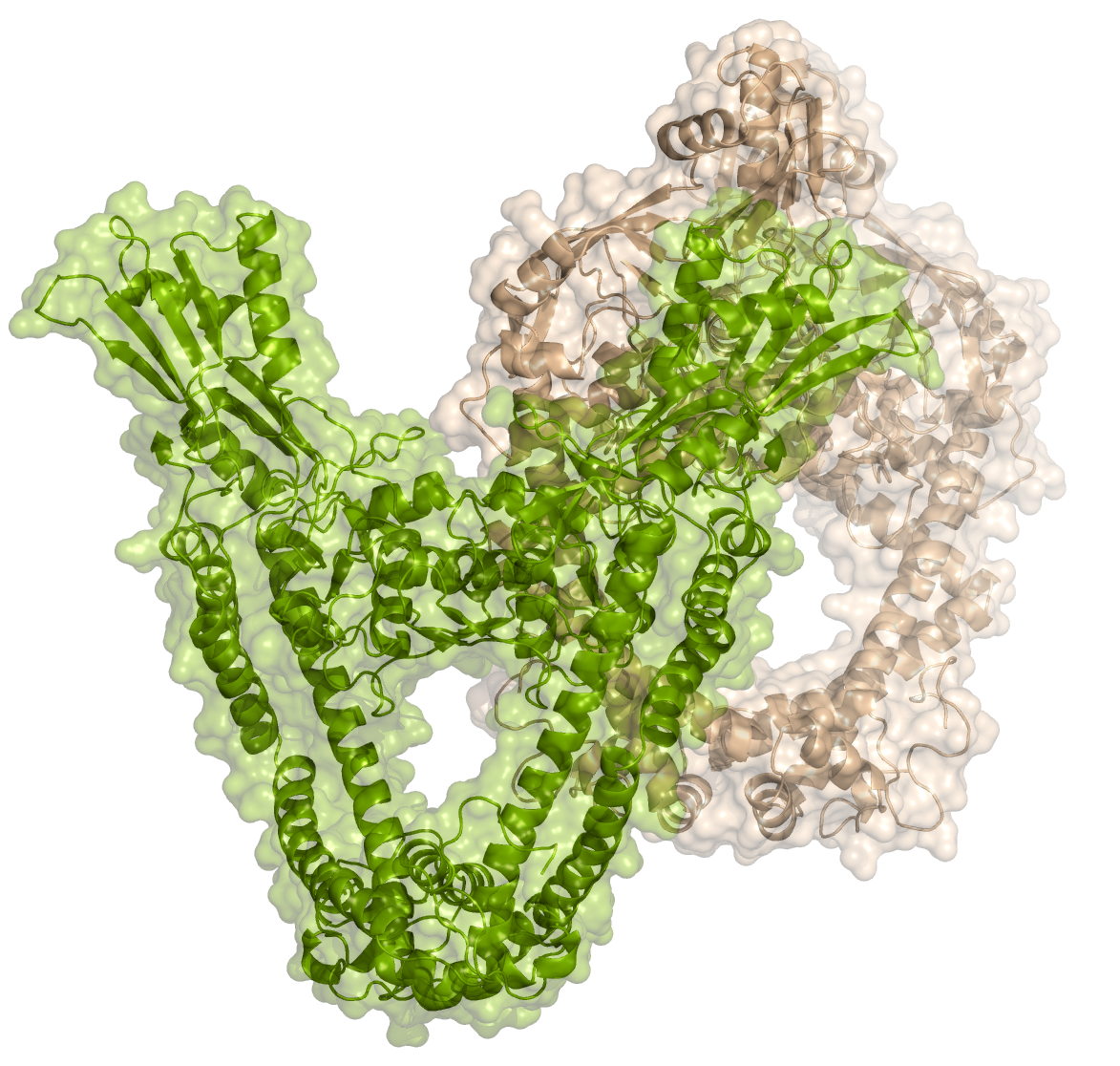


**Fig. S3.** Crystal structure of apoGyrA55, showing the four monomers that comprise the asymmetric unit. The two biological dimers are coloured dark green and light brown, respectively. The protein is depicted in cartoon representation with a semi-transparent surface.

**Figure S4**


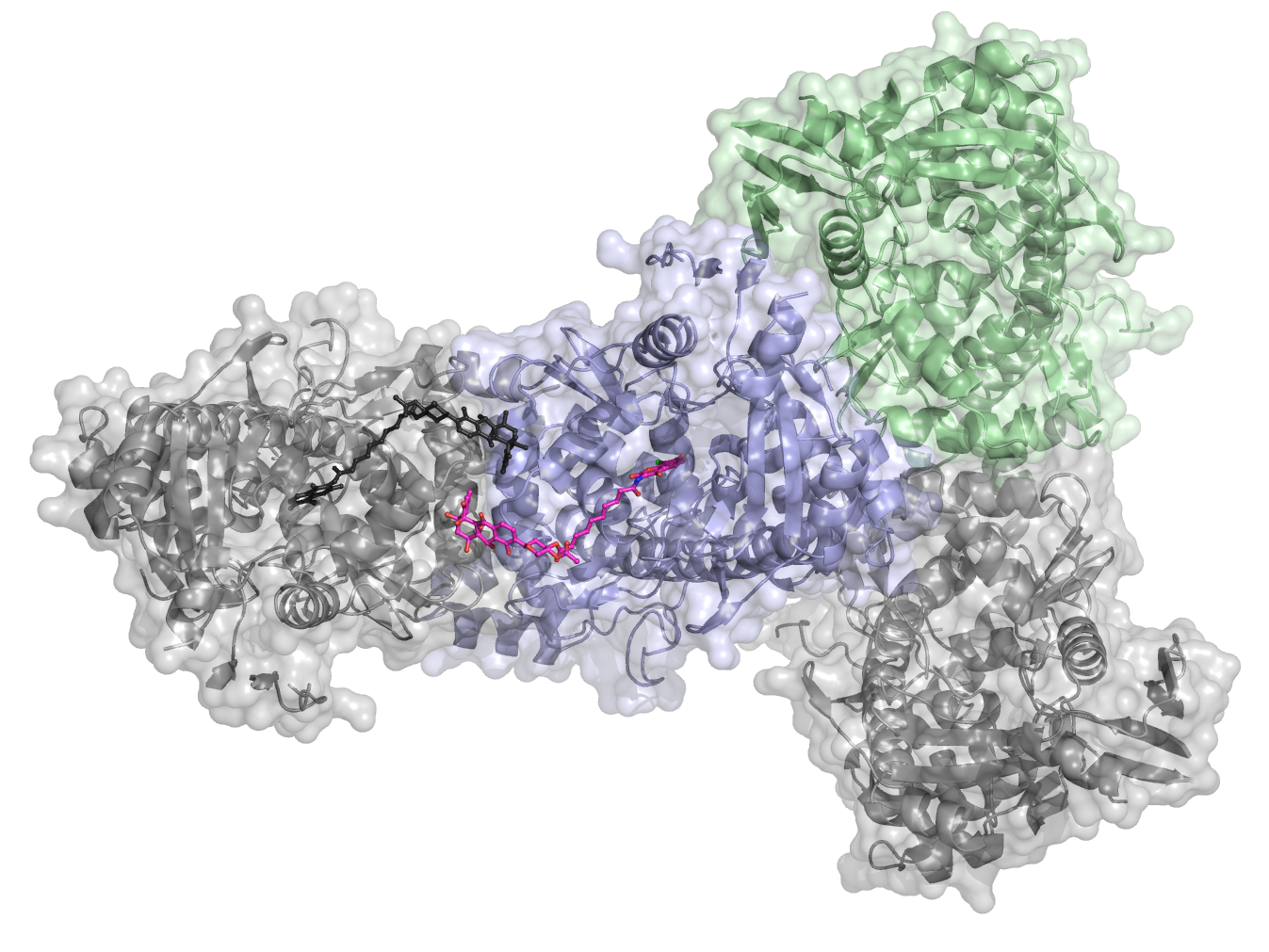


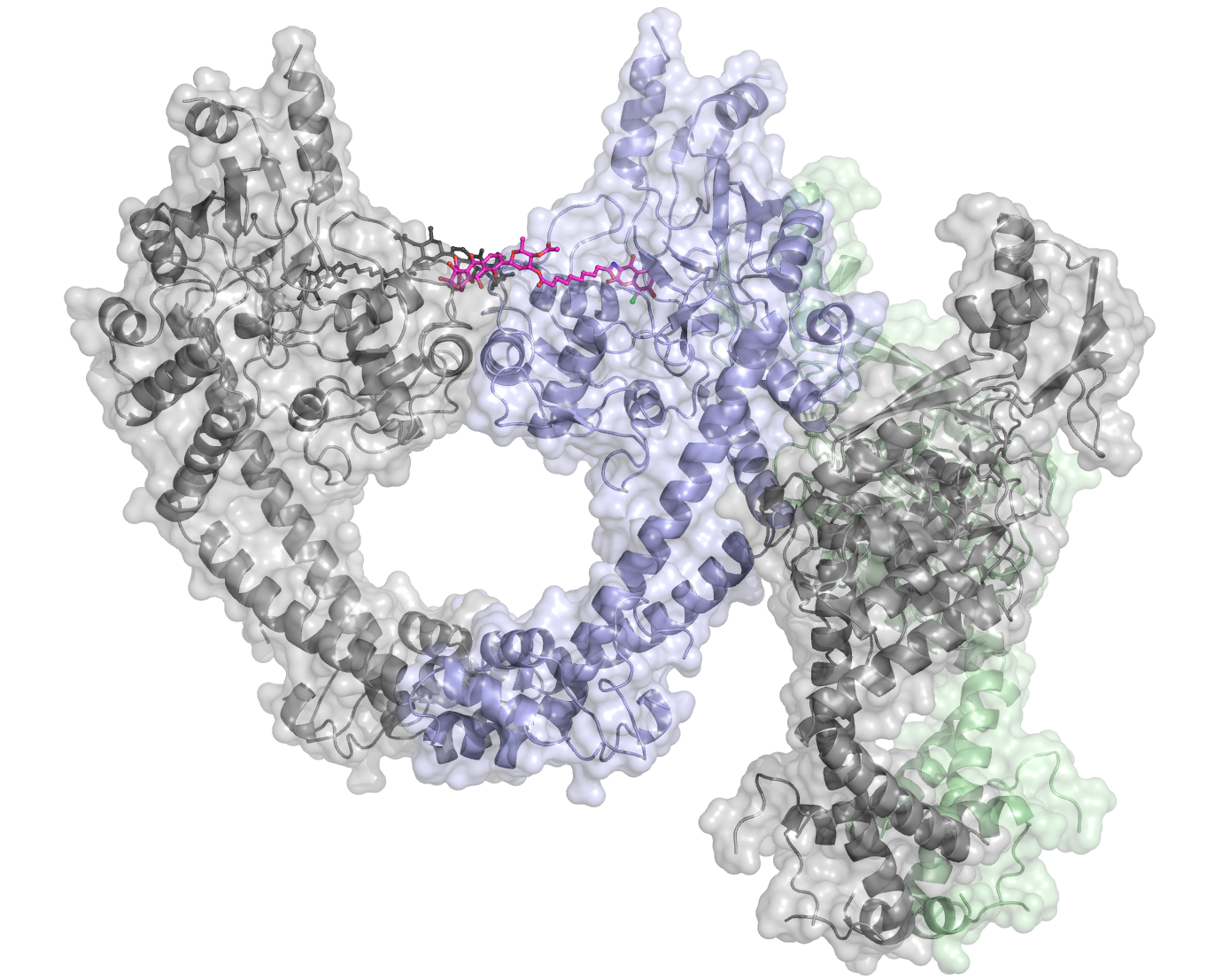


**Fig. S4.**  Orthogonal views of the crystal structure of the GyrA55-SD8 complex, showing the two monomers that comprise the asymmetric unit (blue and green) and the symmetry-related other halves of the biological units (grey). The protein is depicted in cartoon representation with a semi-transparent surface and the SD8 molecules bound to only one subunit are shown as magenta or black sticks in the ASU or symmetry related monomers, respectively.

**Figure S5**

**(a)**

**
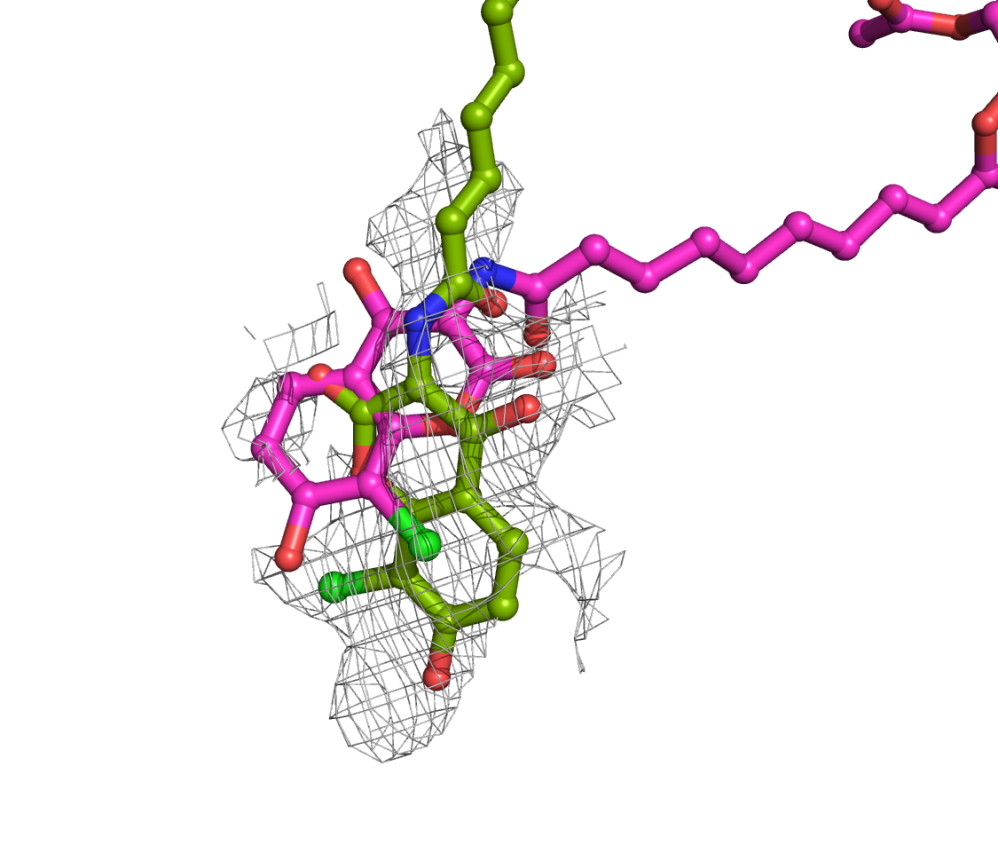
**

**(b)**

**
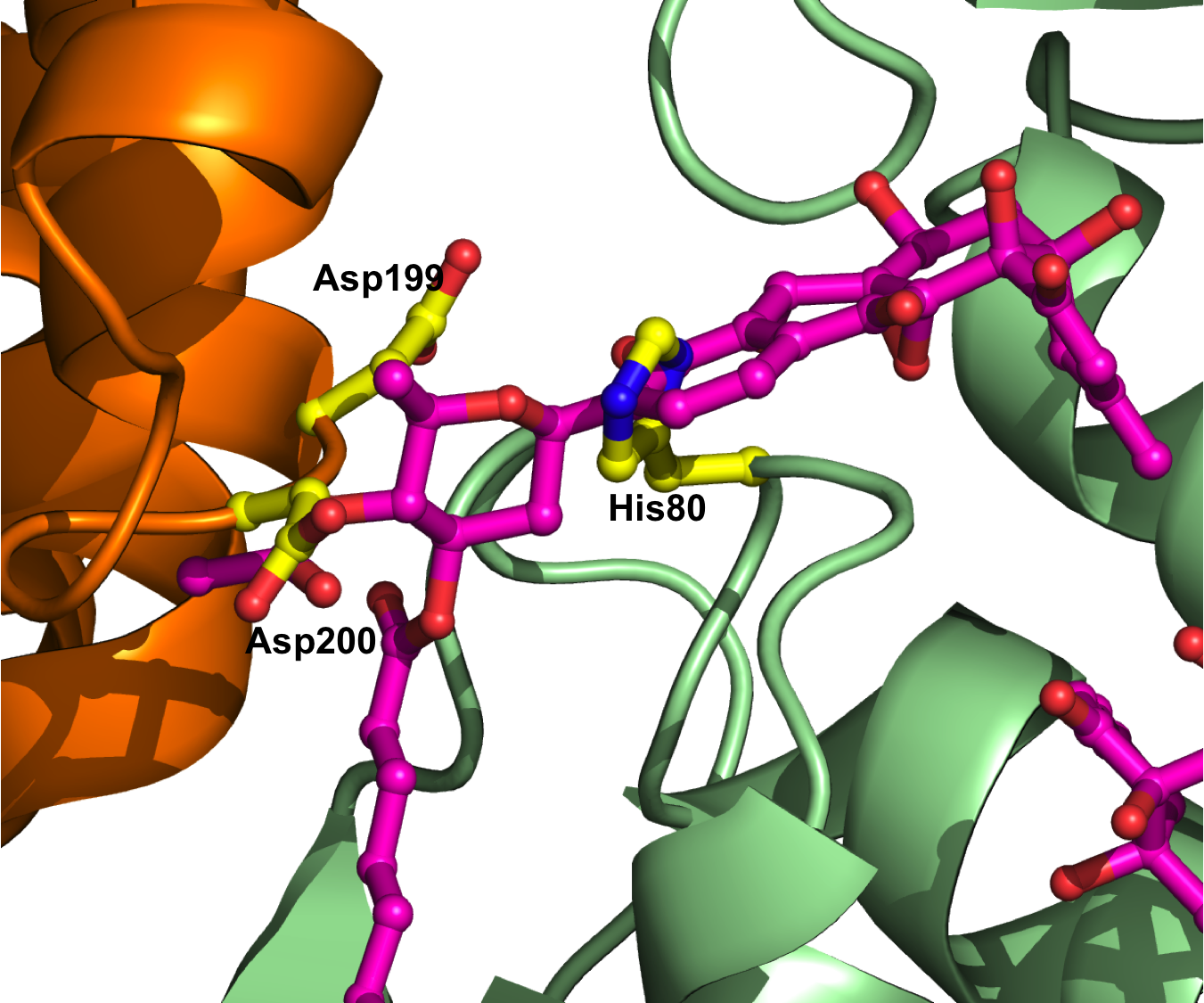
**

**Fig. S5. (a)** Simulated annealing omit electron density in the aminocoumarin-binding pocket of the second biological dimer of the GyrA55-SD8 complex. The first dimer of the same structure was then superposed on this dimer, and the resultant position of the bound SD8 ligand is shown (magenta). Next, the biological dimer of the GyrA59-SD8 complex was superposed on this dimer, and the resultant position of the bound SD8 ligand is shown (green). Thus, this residual electron density is more consistent with the orientation of the aminocoumarin moiety of the SD8 molecule bound to the GyrA59 dimer; the electron density extends partway into the linker, but there is no evidence of density for the polyketide moiety. **(b)** Crystal packing prevents SD8 from binding fully to the second biological dimer of the GyrA55-SD8 complex. The second dimer is shown in green together with the SD8 molecule from the first dimer after superposing on the second dimer. Shown in orange is the true position of the first dimer within the crystal lattice. It is clear that this mode of SD8 binding to dimer 2 would result in steric clashes between dimer 1 and the olivose moiety. In addition, the conformation of His80 in dimer 2 is incompatible with this mode of SD8 binding.

**Figure S6**


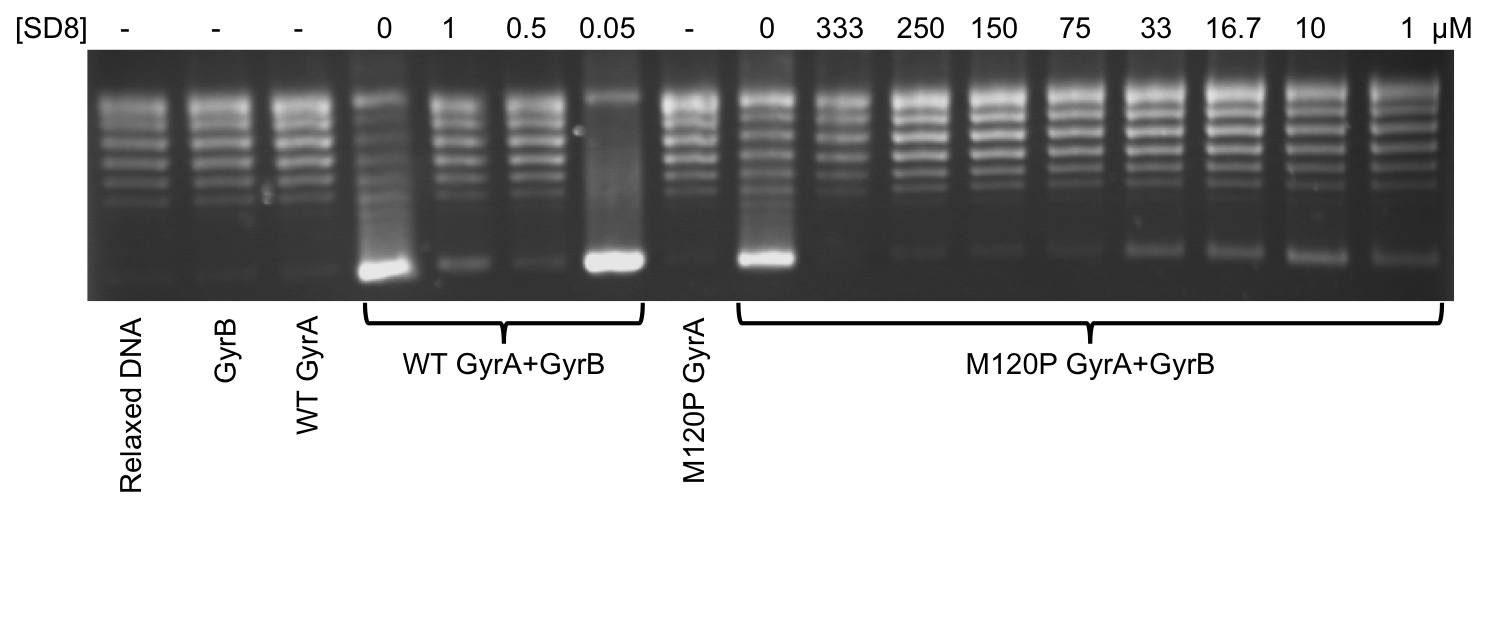


**Fig. S6.** The effect of SD8 on DNA supercoiling by wild type and M120P mutant gyrases. Gyrase (22 nM) and relaxed pBR322 DNA (6 nM) were incubated with decreasing concentrations of SD8 at 37°C for 30-90 minutes depending on the GyrA used: 90 minutes for the less active M120P GyrA mutant; 30 minutes for WT GyrA.
